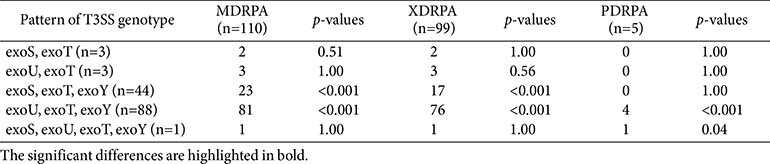# Coexistence of Multidrug Resistance and Hypervirulence-Associated Genes in Clinical Carbapenem-Resistant *Pseudomonas aeruginosa*

**DOI:** 10.4014/jmb.2505.05031

**Published:** 2025-09-24

**Authors:** Hye Hyun Cho, Yumi Park

**Affiliations:** 1 Department of Biomedical Laboratory Science, Daejeon Institute of Science and Technology, Daejeon 35408, Republic of Korea; 2 Department of Laboratory Medicine, Chungnam National University School of Medicine, Chungnam National University Hospital, Daejeon 35015, Republic of Korea

**Keywords:** Carbapenem resistance, multidrug resistance, virulence factor, sequence type, multilocus sequence typing

## Abstract

The recent worldwide emergence of hypervirulent, multidrug-resistant *Pseudomonas aeruginosa* represents a critical public health threat. The molecular typing, multidrug resistance (MDR) rates, and hypervirulence-associated genes in carbapenem-resistant *P. aeruginosa* (CRPA) isolates can vary across geographic locations and patients, highlighting their clinical significance. In this study we investigated the epidemiology and relationship between MDR and the presence of hypervirulence-associated genes in CRPA isolates. Accordingly, we performed antimicrobial susceptibility tests, multilocus sequence typing (MLST), and PCR-based detection of carbapenemase and virulence-associated genes. Notably, 34.9% (139/398) of the isolates were carbapenem-resistant, with 79.1% of these exhibiting MDR. Of the 30 sequence types (STs) identified by MLST, ST773 was the most prevalent (28.8%), followed by ST235 (23.0%). New Delhi metallo-β-lactamase (NDM)-1 or imipenemase-6 occurred in 45.3% of the CRPA strains. Common virulence genes included *exoT* (100.0% of isolates), *exoY* (95.7%), *exoU* (66.2%), and *exoS* (34.5%). *exoU* was associated with antibiotic resistance (*p*<0.05), except for carbapenems in CRPA isolates. The *exoU*/*exoT*/*exoY* Type III secretion system (T3SS) genotype was the most prevalent among the carbapenemase-producing CRPA strains. Among the 139 CRPA isolates, we identified a pandrug-resistant, NDM-1-producing ST235 strain co-expressing *exoS* and *exoU*. This study highlights the important association between *exoU* and MDR, indicating a potential relationship between T3SS genotypes and antibiotic resistance. Additionally, the identification of a hypervirulent, pandrug-resistant *P. aeruginosa* ST235 isolate harboring *bla*_IMP-6_ underscores the critical need for enhanced surveillance against high-risk strains.

## Introduction

*Pseudomonas aeruginosa* is an opportunistic, nosocomial pathogen that causes various healthcare-associated infections in the respiratory tract, urinary tract, skin, soft tissues, and bloodstream [1]. Carbapenems are commonly used in the final treatment of multidrug-resistant *P. aeruginosa* (MDRPA) infections owing to their broad-spectrum antibacterial activity [2]. However, the development of carbapenem resistance poses a major challenge due to the lack of alternative antimicrobial treatments [3].

*P. aeruginosa* commonly acquires carbapenem resistance via the production of carbapenemase [4]. Based on the hydrolytic mechanisms at their active sites, carbapenemases are grouped into classes A, B, and D using the Ambler classification [5]. Class B carbapenemases are the most commonly produced by *P. aeruginosa* clinical isolates.

These include metallo-β-lactamases (MBLs), such as New Delhi metallo-β-lactamases (NDM), imipenemase (IMP), and Verona integron-encoded metallo-β-lactamase (VIM) [6]. Since the carbapenemase produced by P. aeruginosa inactivates carbapenems and antipseudomonal drugs such as ceftazidime and cefepime, as well as more recently introduced treatment agents, including ceftazidime-avibactam, ceftolozane-tazobactam, and imipenem-relebactam, the emergence of carbapenemase-producing *P. aeruginosa* represents an urgent concern [7]. Moreover, the natural resistance of *P. aeruginosa* to various antibiotics and its ability to acquire resistance has given rise to MDR and greatly contributed to morbidity and mortality rates [8].

*P. aeruginosa* has several virulence mechanisms, such as toxin secretion, quorum sensing, and biofilm formation, all of which enhance its infectivity [9]. Carbapenem-resistant *P. aeruginosa* (CRPA), particularly strains carrying hypervirulence-associated genes, pose a considerable threat to public health. A significant determinant of virulence is the Type III secretion system (T3SS), which disrupts host defenses by injecting toxin effectors such as ExoS, ExoU, ExoT, and ExoY into host cells [10, 11]. These effectors subsequently alter the intracellular environment, preventing phagocytosis and bacterial clearance [10, 11]. ExoS and ExoT are bifunctional cytotoxins with GTPase and ADP-ribosyl transferase activities that disrupt the actin cytoskeleton and trigger apoptosis [12]. ExoU, a potent phospholipase, is the most virulent effector, and leads to rapid cell necroptosis [13]. ExoY significantly increases cAMP, cGMP, and cUMP levels in host cells via adenylate or nucleotidyl cyclase activity while reducing inflammatory cytokine production [13]. Under a T3SS genotype, most *P. aeruginosa* strains carry *exoS* or *exoU* owing to the frequent mutual exclusion of the two genes [14]. However, several recent sporadic reports of hypervirulent *P. aeruginosa* strains co-expressing T3SS effectors *exoS* and *exoU*, primarily from clinical origins, have suggested their possible dissemination [14, 15]. The co-existence of MDR and hypervirulence factors in CRPA represents a complex clinical challenge that requires immediate attention [16].

The molecular typing, MDR rate, and distribution of hypervirulence-associated genes in CRPA isolates may vary depending on geographic location, local hospital settings, and the patient population. Although the identification of MDR and hypervirulence-associated genes in clinical CRPA isolates directly affects public health and clinical treatment approaches, no related comprehensive study exists. In this study, we therefore sought to determine the epidemiological characteristics of clinical CRPA isolates using multilocus sequence typing (MLST) and the potential association between the resistance profiles and hypervirulence-associated factors.

## Materials and Methods

### Bacterial Isolates

A total of 398 *P. aeruginosa* isolates were collected from Konyang University Hospital in South Korea between April 2020 and March 2021. The isolates were identified using matrix-assisted laser desorption/ionization time-of-flight mass spectrometry (MALDI-TOF MS; Biotyper, Bruker Daltonics, Germany). Clinical data associated with each isolate were retrospectively reviewed.

### Antimicrobial Susceptibility Testing

Antimicrobial susceptibility tests were conducted using an automated MicroScan WalkAway-96 Plus system (Beckman Coulter, USA) based on the broth microdilution method. The minimum inhibitory concentrations (MICs) were interpreted according to the Clinical and Laboratory Standards Institute guidelines [17]. *P. aeruginosa* ATCC 27853 was used as the quality control strain for each batch of MIC tests. Antimicrobial categories were classified as aminoglycosides (amikacin, tobramycin), carbapenems (imipenem, meropenem), cephalosporins (ceftazidime, cefepime), penicillin with β-lactamase inhibitors (piperacillin/tazobactam), fluoroquinolones (ciprofloxacin, levofloxacin), and monobactams (aztreonam). The antimicrobial-resistant *P. aeruginosa* strains were categorized as follows: 1) CRPA when resistant to imipenem and/or meropenem; 2) MDRPA when resistant to at least one agent in three or more antimicrobial categories; 3) extensively drug-resistant *P. aeruginosa* (XDRPA) when not susceptible to at least one agent in all but two or fewer antimicrobial categories; and 4) pandrug-resistant *P. aeruginosa* (PDRPA) when resistant to all antimicrobial categories [18, 19].

### MLST

The epidemiological relatedness among the CRPA isolates was assessed using MLST based on the sequences of seven housekeeping genes (*acsA*, *aroE*, *guaA*, *mutL*, *nuoD*, *ppsA*, and *trpE*) [20]. Polymerase chain reaction (PCR) amplification was performed on a C1000 Thermal Cycler (Bio-Rad Laboratories, Hercules, CA, USA). The PCR products were sequenced with a 3730xL DNA Analyzer (Applied Biosystems, USA). Sequence types (STs) were assigned using PubMLST (http://pubmlst.org/paeruginosa/) [21].

### Detection of Virulence Factors and Carbapenemase Genes

All CRPA isolates were analyzed for hypervirulence-associated (including *exoS*, *exoU*, *exoT*, and *exoY*) and carbapenemase genes (*bla*_KPC_, *bla*_IMP_, bla_VIM_, bla_NDM_, *bla*_GES_, and *bla*_OXA-48_-like) using PCR as previously described [22-25]. The primers used are listed in Table 1.

### Statistical Analysis

Data analysis was performed using SPSS 26.0 (IBM SPSS, USA). Categorical variables were analyzed using Chi-square or Fisher’s exact test and presented as numbers and percentages. The correlation of *exoS* and *exoU* virulence gene presence with a pattern of antibiotic resistance in CRPA isolates was first evaluated by univariate logistic regression. To assess potential confounding by clonal lineage and specimen source, we fitted a multivariate logistic model including genotype (*exoS*+/*exoU*– vs. *exoS*–/*exoU*+), MLST sequence type, and specimen type as covariates. Statistical significance was set at *p* < 0.05.

## Results

### Clinical CRPA Isolates

We collected 398 nonrepetitive *P. aeruginosa* clinical isolates, of which 139 (34.9%) were CRPA and 259 (65.1%) were non-CRPA. The CRPA isolates originated from various clinical specimens, including urine (43.2%, 60/139), sputum (37.4%, 52/139), deep-wound swab (7.9%, 11/139), abscess (2.9%, 4/139), blood (2.9%, 4/139), bile (2.2%, 3/139), ear swab (1.4%, 2/139), peritoneal fluid (1.4%, 2/139), and catheter tip (0.7%, 1/139), and different hospital wards, including pulmonology (26.6%, 37/139), nephrology (17.3%, 24/139), urology (12.9%, 18/139), emergency medicine (7.2%, 10/139), neurosurgery (5.8%, 8/139), general surgery (5.8%, 8/139), orthopedic surgery (5.8%, 8/ 139), hemato-oncology (4.3%, 6/139), plastic surgery (2.9%, 4/139), cardiology (2.2%, 3/139), otorhinolaryngology (2.2%, 3/139), rehabilitation medicine (2.2%, 3/139), gastrointestinal medicine (1.4%, 2/139), cardiac surgery (1.4%, 2/139), infectious diseases (0.7%, 1/139), pediatric (0.7%, 1/139), and neurology (0.7%, 1/139) (Table 2). No significant association was observed between clinical samples and carbapenem resistance, except for ear swabs (*p* = 0.01) and urine (*p* < 0.001). The median age of patients was 74 (range, 14–97) years, and 67.6% (94/139) and 32.4% (45/139) of the CRPA isolates were obtained from males and females, respectively.

### Antibiotic and Multidrug Resistance Profiles

**Fig. 1:**
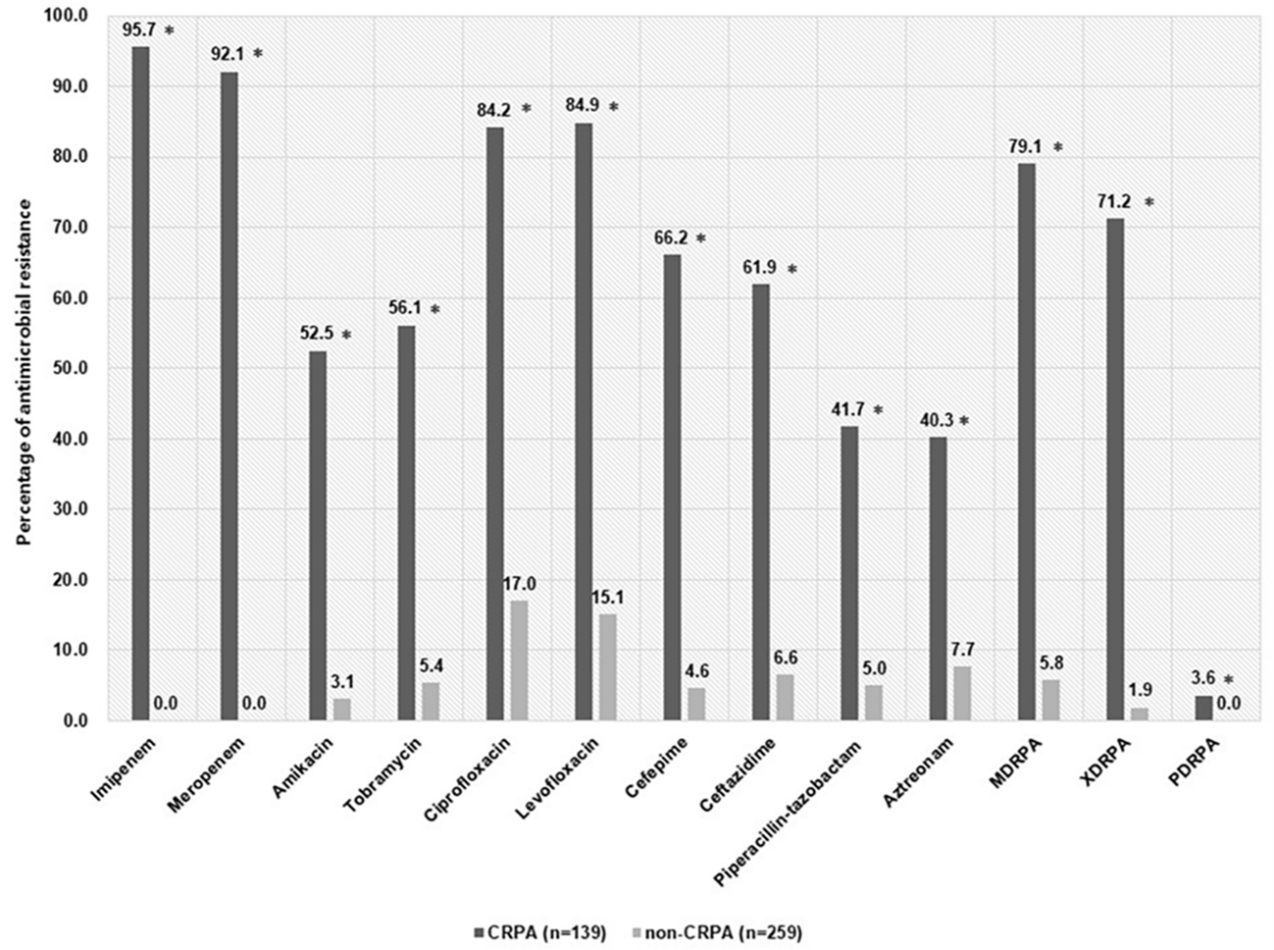
Prevalence of resistance to various antibiotics among 139 carbapenem-resistant *P. aeruginosa* (CRPA) and 259 non-carbapenem-resistant *P. aeruginosa* isolates (non-CRPA). The *p*-values indicate significance where *p<0.05 (significant). MDRPA, multidrug-resistant *P. aeruginosa*; XDRPA, extensively drug-resistant *P. aeruginosa*; PDRPA, pandrug-resistant *P. aeruginosa*.

The antimicrobial resistance profiles of the 139 CRPA and 259 non-CRPA isolates are illustrated in Fig. 1. The CRPA isolates were resistant to imipenem (95.7%, 133/139), meropenem (92.1%,128/139), levofloxacin (84.9%, 118/139), ciprofloxacin (84.2%, 117/139), cefepime (66.2%, 92/139), ceftazidime (61.9%, 86/139), tobramycin (56.1%, 78/139), amikacin (52.5%, 73/139), piperacillin-tazobactam (41.7%, 58/139), and aztreonam (40.3%, 56/ 139). The non-CRPA isolates were resistant to ciprofloxacin (17.0%, 44/259), levofloxacin (15.1%, 39/259), aztreonam (7.7%, 20/259), ceftazidime (6.6%, 17/259), tobramycin (5.4%, 14/259), piperacillin-tazobactam (5.0%, 13/259), cefepime (4.6%, 12/259), and amikacin (3.1%, 8/259). The CRPA strains exhibited higher resistance rates to all antimicrobials than the non-CRPA isolates (*p* < 0.05). Among the CRPA isolates, MDRPA, XDRPA, and PDRPA were identified in 79.1% (110/139), 71.2% (99/139), and 3.6% (5/139) of cases, respectively.

### Molecular Epidemiology Based on MLST and Carbapenemase Gene Distribution

MLST revealed 30 STs for the CRPA isolates, namely ST773 (28.8%, 40/139), ST235 (23.0%, 32/139), ST2238 (10.8%, 15/139), ST357 (5.8%, 8/139), ST446 (5.0%, 7/139), ST17 (2.9%, 4/139), ST111 (2.2%, 3/139), ST244 (2.2%, 3/139), ST245 (2.2%, 3/139), ST1031 (1.4%, 2/139), ST1248 (1.4%, 2/139), ST1568 (1.4%, 2/139), ST27 (0.7%, 1/139), ST132 (0.7%, 1/139), ST168 (0.7%, 1/139), ST207 (0.7%, 1/139), ST253 (0.7%, 1/139), ST266 (0.7%, 1/139), ST274 (0.7%, 1/139), ST277 (0.7%, 1/139), ST298 (0.7%, 1/139), ST480 (0.7%, 1/139), ST508 (0.7%, 1/ 139), ST709 (0.7%, 1/139), ST1215 (0.7%, 1/139), ST1341 (0.7%, 1/139), ST1455 (0.7%, 1/139), ST1682 (0.7%, 1/ 139), ST1756 (0.7%, 1/139), and ST2316 (0.7%, 1/139). The carbapenemases were detected in 45.3% (63/139) of the CRPA isolates. NDM-1 (57.1%, 36/63) was the most prevalent carbapenemase, followed by IMP-6 (42.9%, 27/ 63), whereas *bla*_KPC_, bla_VIM_, *bla*_GES_, and *bla*_OXA-48_-like were not detected in the CRPA isolates. The CRPA isolates harboring *bla*_IMP-6_ and *bla*_NDM-1_ were associated with ST235 and ST773, respectively (Table 3). Consistently, 84.4% of ST235 and 90.0% of ST773 were carbapenemase-producing CRPA isolates. All CRPA isolates belonging to ST111, -132, -168, -235, -245, -253, -266, -508, -709, -773, -1215, -1248, and -1455 were confirmed to have MDR.

### Distribution of Virulence Factors

Regarding the prevalence of T3SS-related genes in the CRPA isolates, the most common virulence gene was *exoT* (100.0%, 139/139), followed by *exoY* (95.7%, 133/139) and *exoU* (66.2%, 92/139), while *exoS* was the least common (34.5%, 48/139). Among the CRPA isolates, the most frequently observed T3SS genotype combination was *exoU*/*exoT*/*exoY* (63.3%, 88/139), followed by *exoS*/*exoT*/*exoY* (31.7%, 44/139), *exoS*/*exoT* (2.2%, 3/139), *exoU*/*exoT* (2.2%, 3/139), and *exoS*/*exoU*/*exoT*/*exoY* (0.7%, 1/139). All CRPA isolates expressed *exoS*–/*exoU*+ (65.9%, 91/138) or *exoS*+/*exoU*– (34.1%, 47/138), except for one strain carrying *exoS* and *exoU* concurrently. With the exception of the latter isolate, 62 carbapenemase-producing CRPA isolates exhibited the *exoS*–/*exoU*+ genotype, with the most common combination being *exoU*/*exoT*/*exoY* (96.8%, 60/62), followed by *exoU*/*exoT* (3.2%, 2/62). The *exoS*–/*exoU*+ genotype was identified in CRPA ST207, -235, -253, -298, -357, -446, -773, and -1248, while the *exoS*+/*exoU*– genotype was identified in CRPA ST17, -27, -111, -132, -168, -244, -245, -266, -274, -277, -480, -508, -709, -1031, -1215, -1341, -1455, -1568, -1682, -1756, -2238, and -2316. The CRPA isolate co-expressing *exoS* and *exoU* was a PDR NDM-1-producing ST235 strain. In a univariate correlation analysis between the *exoS*+/*exoU*– and *exoS*-/*exoU*+ genotypes and antibiotic resistance of CRPA isolates, the presence of *exoU* was significantly associated with resistance to aminoglycosides, cephalosporins, penicillin+β-lactamase inhibitor, fluoroquinolones, and monobactam (all *p* < 0.05) (Table 4). However, multivariate logistic regression models adjusted for the sequence and specimen types indicated that the *exoS*–/*exoU*+ genotype was not significantly associated with resistance to any of the antibiotic classes tested (Table S1). MDRPA, PDRPA, and XDRPA were related to the *exoU*/*exoT*/*exoY* virulence genotype (*p* < 0.001) (Table 5).

## Discussion

Molecular epidemiological studies on the relationship between virulence genotypes and resistance phenotypes are crucial for elucidating the epidemiological characteristics of *P. aeruginosa* infections at the genetic level. Similarly, understanding the molecular typing and antibiotic resistance patterns of CRPA is essential for optimizing antibiotic therapy and improving infection control strategies.

In this study, 34.9% of *P. aeruginosa* isolates were resistant to carbapenems, falling between the global range of 10% to 70% [26, 27]. Except for ear swab and urine samples, clinical specimen types were not significantly associated with carbapenem resistance. CRPA isolates exhibited significantly higher resistance rates to antibiotics than the non-CRPA isolates. Furthermore, 79.1% of CRPA strains were classified as MDR, notably higher than the previously reported 62–68% [28-30]. CRPA isolates were also more likely to be categorized as MDRPA, XDRPA, and PDRPA than non-CRPA isolates.

Carbapenem resistance in *P. aeruginosa* is attributed to various mechanisms, including carbapenemase production, reduced membrane permeability, and efflux pump overexpression [31]. The prevalence of specific carbapenemase-encoding genes may differ based on geographic distribution and endemic factors [32]. This study showed that 45.3% of CRPA isolates carried carbapenemase genes, with the most common being *bla*_NDM-1_, followed by *bla*_IMP-6_, consistent with previous reports in the Republic of Korea [33-35].

Furthermore, MLST analysis of 139 CRPA isolates revealed 30 distinct STs. The *P. aeruginosa* clones ST111, ST244, ST235, ST277, ST298, and ST357 are globally widespread, frequently associated with MDR, and considered "international" or "high-risk" clones [36]. Notably, 84.4% of ST235 and 90.0% of ST773 isolates produced carbapenemases, indicating their strong association with carbapenem resistance. ST235 predominantly harbored the *bla*_IMP-6_ gene, while ST773 strains carried *bla*_NDM-1_, consistent with previous studies reporting lineage-specific carbapenemase gene distributions [33-35].

The high antibiotic resistance rate of CRPA isolates poses a major clinical challenge, particularly when they co-harbor hypervirulence-associated genes. Identifying virulence genes is critical for developing effective treatment strategies [16]. In this study, we analyzed the prevalence of hypervirulence-associated genes (*exoS*, *exoU*, *exoT*, and *exoY*) in CRPA isolates and found that *exoT* (100.0%) and *exoY* (95.7%) predominated, followed by *exoU* (66.2%) and *exoS* (34.5%). This pattern aligns with previous reports, except for the higher prevalence of *exoU* compared to *exoS* [16, 32, 37-40]. This discrepancy may be attributed to the dominance of *exoU*-carrying clones, underscoring the importance of regional or strain-specific variations in the distribution of T3SS effector genes.

Because the presence of *exoS* and *exoU* is generally mutually exclusive, the *exoS*+/*exoU*+ genotype is rare [14]. Nevertheless, previous studies have reported isolates harboring both genes, and some *P. aeruginosa* strains have been identified with the complete T3SS genotype (*exoS*+/*exoU*+/*exoT*+/*exoY*+) [14, 32, 42, 43]. The distribution of ExoS and ExoU cytotoxin genes appears to vary depending on the genetic background of the strain or site of infection [43]. Notably, co-expression and co-secretion of ExoS and ExoU have been shown to enhance both in vitro cytotoxicity and *in vivo* pathogenicity [15].

In our univariate analyses of the CRPA isolates, *exoU* expression was significantly associated with resistance to multiple antibiotics, including amikacin, aztreonam, cefepime, ceftazidime, ciprofloxacin, levofloxacin, and piperacillin-tazobactam. However, these associations were non-significant after adjustment for MLST sequence and specimen types in the multivariate logistic regression. Despite attempts to control for potential confounders, the analysis was fundamentally limited by near-complete collinearity among the *exoU* genotype, sequence, and specimen types. Predominant sequence types, such as ST773 and ST235, were exclusively *exoS*–/*exoU*+, while ST2238 and other minor STs were *exoS*+/*exoU*–. Additionally, most urine (57/59) and sputum (37/52) isolates were *exoS*–/*exoU*+. This structural dependency rendered *exoU* status almost entirely predictable by MLST and specimen type, precluding independent effect estimation within the model. Consequently, the multivariate model failed to disentangle the specific contribution of *exoU* from the clonal background and specimen characteristics. This limitation highlights the inherent challenge in observational studies of clonal pathogens, where virulence and resistance factors often co-exist within dominant lineages. To mitigate collinearity and accurately assess the independent impact of *exoU* on antimicrobial resistance, future studies should include larger and more genetically diverse isolate collections.

MDRPA, XDRPA, and PDRPA strains were predominantly associated with the *exoU*/*exoT*/*exoY* genotype. This strong association underscores the importance of considering T3SS virulence genotypes when evaluating the resistance profiles of clinical isolates. The *exoU* gene has been frequently identified in MDRPA strains, particularly those resistant to carbapenems and fluoroquinolones [44]. *P. aeruginosa* strains harboring *exoU* have been associated with higher MDR and mortality rates than those harboring other T3SS genes [40, 45, 46].

We characterized the frequency of individual T3SS genes based on the carriage of carbapenemase genes in CRPA strains, and confirmed that carbapenemase-producing CRPA isolates harbored *exoU*. This suggests that *exoU* is especially prevalent in carbapenemase-producing strains, potentially contributing to their virulence and resistance profiles. Additionally, the *exoU*/*exoT*/*exoY* genotype was observed in most carbapenemase-producing strains, while *exoS* was absent from all carbapenemase-producing CRPA strains except one with the *exoS*+/*exoU*+ genotype, indicating a potential incompatibility between these two genes. Previous studies have also shown that *exoS* is relatively less common in CRPA strains harboring the carbapenemase gene [8]. Further investigation with a larger sample size and deeper exploration of the underlying mechanisms is necessary to clarify this relationship.

Consistent with previous results, *exoS*–/*exoU*+ was identified in ST235, -298, -357, and -446, and *exoS*+/*exoU*–was identified in ST17, -27, -111, -244, -245, -274, -277, and -508 [47, 48]. Clones with the *exoS*+/*exoU*– genotype, such as ST111, have been associated with reduced cytotoxicity, invasiveness, and virulence [49].

The fitness costs associated with acquiring resistance mechanisms are generally considered to reduce the virulence of strains with MDR/XDR [10]. However, in this study, we identified a PDRPA ST235 strain harboring both *exoS* and *exoU* within the *exoS*/*exoU*/*exoT*/*exoY* genotype, which also carried the *bla*_IMP-6_ carbapenemase gene.

The *P. aeruginosa* ST235 clone has dispersed globally, particularly in Europe and Asia, and is associated with poor clinical outcomes owing to MDR, including the production of MBLs such as IMP, NDM, and VIM, as well as high virulence due to the presence of *exoU* [50, 51]. Previous studies have also shown that IMP-producing XDRPA ST235 strains co-express *exoU* and *exoS* [14].

This study has some limitations. First, it was a single-center study conducted at a university hospital with a limited regional scope, and thus may not represent the overall characteristics of CRPA isolates in the entire Republic of Korea. Second, although the presence of T3SS-related genes was identified by PCR, functional assays, such as gene expression, protein secretion, and cytotoxicity testing, were not performed. Further studies are needed to determine whether these genes are actively expressed and contribute to virulence in clinical infections. Additionally, while MLST provided useful insights into the clonal distribution of CRPA isolates, it was insufficient to elucidate the genetic mechanisms underlying the co-carriage of resistance and virulence factors. Whole-genome sequencing (WGS) would offer a more comprehensive understanding of the genetic context linking these traits but was outside the scope of the current study due to limited resources. We intend to incorporate comprehensive genomic analyses in future research.

In conclusion, our study elaborates on the epidemiological characteristics and virulence factors of clinical CRPA isolates. The findings highlight the important influence of clonal type and the T3SS genotype on the clinical impact and outcomes of infections by MDR strains. Of particular concern is the identification of a hypervirulent PDRPA ST235 isolate co-harboring *exoS*, *exoU*, and the *bla*_IMP-6_ carbapenemase gene. The emergence of these high-risk clones can substantially exacerbate clinical treatment failure and mortality rates. These findings underscore the urgent need for vigilant molecular surveillance and robust infection control measures to prevent further dissemination of hypervirulent MDRPA strains. Collectively, our study highlights the complex interplay between antimicrobial resistance and virulence in CRPA isolates, and the importance of integrating molecular epidemiology in clinical management to effectively combat infections caused by high-risk clones.

## Supplemental Materials

Supplementary data for this paper are available on-line only at http://jmb.or.kr.



## Figures and Tables

**Table 1:** Primers used to detect the genes of carbapenemase and virulence factors.

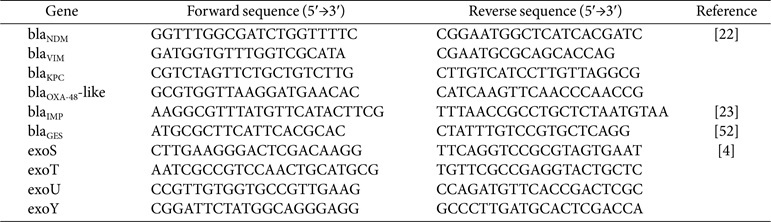

**Table 2:** Distribution of clinical specimens and carbapenem resistance rate between carbapenem-resistant *P. aeruginosa* (CRPA) and non-carbapenem-resistant *P. aeruginosa* (non-CRPA) isolates.

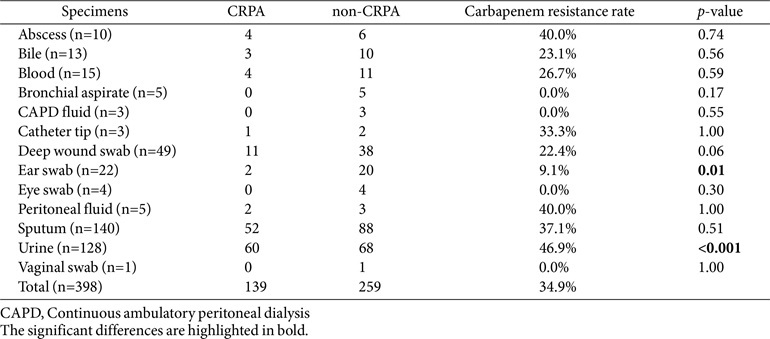

**Table 3:** Distribution of multidrug-resistant *P. aeruginosa* (MDRPA), extensively drug-resistant *P. aeruginosa* (XDRPA), pandrug-resistant *P. aeruginosa* (PDRPA), carbapenemase genes, and hypervirulence-associated genotypes according to sequence type (ST) of carbapenem-resistant *P. aeruginosa* isolates.

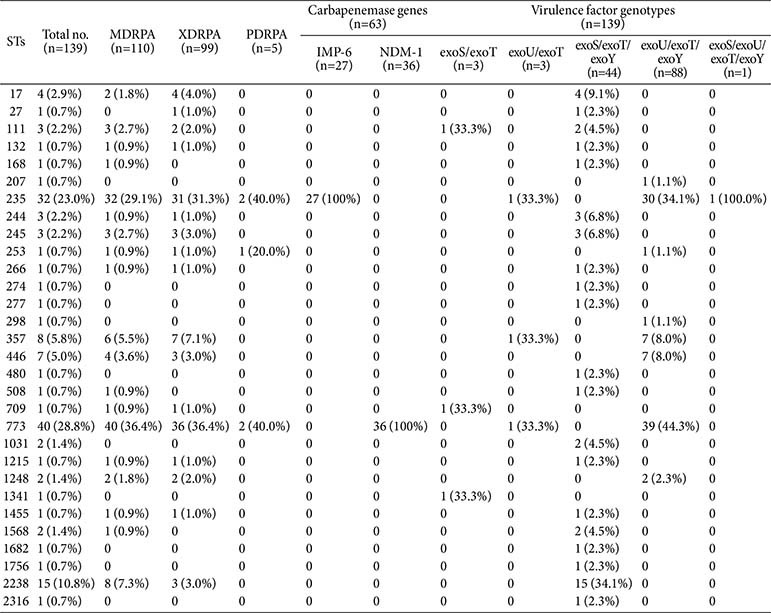

**Table 4:** Correlation of *exoS* and *exoU* virulence genes with antibiotic resistance pattern in carbapenemresistant *P. aeruginosa* isolates except one strain with the *exoS*+/*exoU*+ genotype.

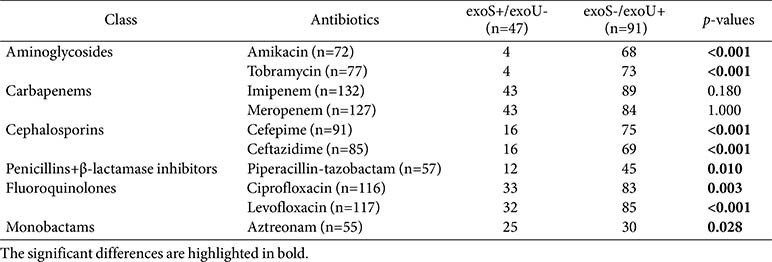

**Table 5:** Distribution of type III secretion system (T3SS) genotype in multidrug-resistant *P. aeruginosa* (MDRPA), extensively drug-resistant *P. aeruginosa* (XDRPA), pandrug-resistant *P. aeruginosa* (PDRPA) isolates.