# Supplementary material for: Coexistence of Multidrug Resistance and Hypervirulence-Associated Genes in Clinical Carbapenem-Resistant Pseudomonas aeruginosa
Source: J Microbiol Biotechnol. 2025 Sep 24;35:e2505031. doi: 10.4014/jmb.2505.05031 (PMC12535857; doi:10.4014/jmb.2505.05031)
Supplement: Supplementary file 1 [file jmb-35-e2505031-supple.pdf]

J. Microbiol. Biotechnol. 2025. 35: e2505031

https://doi.org/10.4014/jmb.2505.05031

Supplementary Table

Table S1. Adjusted associations between *exoS*–/*exoU*+ genotype and antibiotic resistance in carbapenem-resistant *P. aeruginosa* isolates (excluding one *exoS*+/*exoU*+ strain).

Comparison	Class	Agent	Adjusted OR	95% CI	p-value
exoS–/*exoU*+ vs. *exoS*+/*exoU*–	Aminoglycosides	Amikacin	1.70	0.34–8.62	0.522
		Tobramycin	1.51	0.28–8.24	0.635
	Carbapenems	Imipenem	∞ (Not estimable)[1]	—	0.998
		Meropenem	0.50	0.10–2.49	0.397
	Cephalosporins	Cefepime	1.43	0.46–4.44	0.540
		Ceftazidime	1.05	0.34–3.30	0.931
	Penicillin/β-lactamase inhibitors	Piperacillin–tazobactam	1.34	0.41–4.43	0.631
	Fluoroquinolones	Ciprofloxacin	0.97	0.30–3.14	0.957
		Levofloxacin	1.81	0.50–6.59	0.364
	Monobactams	Aztreonam	0.89	0.31–2.55	0.832

[1] Odds ratio infinite due to complete separation; model failed to converge for this comparison. Independent estimation of the *exoU* effect is not feasible due to perfect collinearity.

Model: Multivariable logistic regression; dependent variable = antibiotic resistance (yes/no) to each agent; covariates = MLST sequence type, specimen type. Reference genotype: *exoS*+/*exoU*–.
